# The impact of local and national restrictions in response to COVID-19 on social contacts in England: a longitudinal natural experiment

**DOI:** 10.1186/s12916-021-01924-7

**Published:** 2021-02-19

**Authors:** Christopher I. Jarvis, Amy Gimma, Kevin van Zandvoort, Kerry L. M. Wong, CMMID COVID-19 working group, Kaja Abbas, C. Julian Villabona-Arenas, Kathleen O’Reilly, Matthew Quaife, Alicia Rosello, Adam J. Kucharski, Hamish P. Gibbs, Katherine E. Atkins, Rosanna C. Barnard, Nikos I. Bosse, Simon R. Procter, Sophie R. Meakin, Fiona Yueqian Sun, Sam Abbott, James D. Munday, Timothy W. Russell, Stefan Flasche, Katharine Sherratt, Rosalind M. Eggo, Nicholas G. Davies, Billy J. Quilty, Megan Auzenbergs, Joel Hellewell, Thibaut Jombart, Yalda Jafari, Quentin J. Leclerc, Rachel Lowe, Anna M. Foss, Mark Jit, Arminder K. Deol, Stéphane Hué, Gwenan M. Knight, Akira Endo, Kiesha Prem, Jon C. Emery, Samuel Clifford, Graham Medley, Sebastian Funk, Frank G. Sandmann, Damien C. Tully, Carl A. B. Pearson, Georgia R. Gore-Langton, Alicia Showering, Rein M. G. J. Houben, Emily S. Nightingale, Petra Klepac, Naomi R. Waterlow, Yung-Wai Desmond Chan, James W. Rudge, David Simons, Charlie Diamond, Jack Williams, Oliver Brady, Yang Liu, W. John Edmunds

**Affiliations:** Centre for Mathematical Modelling of Infectious Diseases, Department of Infectious Disease Epidemiology, London School of Hygiene and Tropical Medicine, Keppel Street, London, WC1E 7HT UK

**Keywords:** COVID-19, Contact survey, Lockdowns, Pandemic, Disease outbreak, Non-pharmaceutical interventions, United Kingdom, England

## Abstract

**Background:**

England’s COVID-19 response transitioned from a national lockdown to localised interventions. In response to rising cases, these were supplemented by national restrictions on contacts (the Rule of Six), then 10 pm closing for bars and restaurants, and encouragement to work from home. These were quickly followed by a 3-tier system applying different restrictions in different localities. As cases continued to rise, a second national lockdown was declared. We used a national survey to quantify the impact of these restrictions on epidemiologically relevant contacts.

**Methods:**

We compared paired measures on setting-specific contacts before and after each restriction started and tested for differences using paired permutation tests on the mean change in contacts and the proportion of individuals decreasing their contacts.

**Results:**

Following the imposition of each measure, individuals tended to report fewer contacts than they had before. However, the magnitude of the changes was relatively small and variable. For instance, although early closure of bars and restaurants appeared to have no measurable effect on contacts, the work from home directive reduced mean daily work contacts by 0.99 (95% confidence interval CI] 0.03–1.94), and the Rule of Six reduced non-work and school contacts by a mean of 0.25 (0.01–0.5) per day. Whilst Tier 3 appeared to also reduce non-work and school contacts, the evidence for an effect of the lesser restrictions (Tiers 1 and 2) was much weaker. There may also have been some evidence of saturation of effects, with those who were in Tier 1 (least restrictive) reducing their contacts markedly when they entered lockdown, which was not reflected in similar changes in those who were already under tighter restrictions (Tiers 2 and 3).

**Conclusions:**

The imposition of various local and national measures in England during the summer and autumn of 2020 has gradually reduced contacts. However, these changes are smaller than the initial lockdown in March. This may partly be because many individuals were already starting from a lower number of contacts.

**Supplementary Information:**

The online version contains supplementary material available at 10.1186/s12916-021-01924-7.

## Background

On the 23rd of March 2020, England, along with the rest of the United Kingdom (UK) went into a national lockdown in response to COVID-19 [1]. This required people to only leave their house for essential shopping or medical needs, or to undertake one form of exercise per day. Educational establishments and non-essential retail were closed, as were the leisure and hospitality sectors [1]. Many European countries also implemented national lockdowns and the combinations of large-scale restrictions resulted in marked decreases in contacts, mobility, and transmission, eventually leading to a reduction in daily cases and deaths [2–5].

As the incidence of cases declined, national restrictions were relaxed [5]. England transitioned to a localised response and only applied more stringent restrictions to specific areas with rising cases. The first of these local measures was announced on the 29th of June in Leicester [6], then subsequently in other areas, mostly in the North of England [7]. Local restrictions vary in magnitude but may include early business closures, take-away services only for bars and restaurants, bans on meeting with other households, and travel restrictions.

Alongside local restrictions, in response to rising cases, several national measures were also introduced. On the 14th of September, the *Rule of Six* was announced in England preventing individuals from meeting in groups with more than six people [8]. On the 24th of September, it was announced that pubs and restaurants would be required to close at 10 pm and individuals were encouraged to work from home [9]. Cases continued to rise, and the government combined several restrictions to create a three-tier system ranging from Tier 1 (medium risk) to Tier 3 (very high risk). This tier system was then followed by a second English lockdown from November 5th to December 2nd [10, 11].

The impact of the less severe measures remains unclear, with cases continuing to rise in most localities after measures were implemented. Picking up (perhaps modest) changes in cases, hospitalisations, or deaths some time after restrictions are introduced would be expected to be difficult. In this paper, we avoid these problems by using repeated measures of individuals’ epidemiologically relevant setting-specific contacts before and after restrictions were imposed to estimate whether these measures had any effect and if so their magnitude.

## Methods

### Ethics statement

Participation in this opt-in study was voluntary, and all analyses were carried out on anonymised data. The study was approved by the ethics committee of the London School of Hygiene & Tropical Medicine Reference number 21795.

### Data

We combined data from the English participants of the UK CoMix survey and information on local and national restrictions from Gov.uk. Details of the CoMix study including the protocol and survey instrument have been published previously [2]. In short, CoMix is an online survey where individuals record details of all their direct (i.e. potentially risky) contacts in the day prior to the survey. A direct contact was defined as anyone who was met in person and with whom at least one word was exchanged, or anyone with whom the participants had any sort of skin-to-skin contact. Contacts of individuals under the age of 18 were collected by asking parents to answer on behalf of their child. Information is collected weekly from two alternating, broadly representative, panels (each about 2500 people in size), with each person surveyed once every 2 weeks.

We extracted the start and end dates of restrictions and their locations from Gov.uk between August 31st and December 7th, 2020. CoMix participants were considered affected by local restrictions if they reported living within a Lower Tier Local Authority (UK administrative zone) that was under different restrictions from those applied nationally. We restricted the data to 16 days before and after each restriction came into place to allow for two full weeks of survey responses. We then extracted the closest survey response before and after each restriction date. Participants with missing survey responses either side of the start of a restriction were removed, giving two records per person.

### Details of restrictions

Local restrictions included a range of rules that were inconsistently applied across regions. Most local restrictions fell into four categories: travel restrictions, non-essential closures, preventing indoor mixing, and discouraging overnight stays. Travel restrictions included any of essential travel only, travel being discouraged, and residents banned from leaving their local area. Non-essential closures included places of worship, non-essential retail, gyms, public buildings, personal care services, art venues, and tourist attractions [7].

The Rule of Six prevented individuals from meeting in groups of six or more indoors and outdoors. The 10 pm closure stipulated that hospitality venues must be closed with customers having left the premises by 10 pm. Work from home relates to individuals being encouraged to work from home where possible.

The three-tier system was created on the 14th of October, each tier built upon the previous tier with Tier 1 being the least stringent and Tier 3 the most [10]. Tier 1 (medium risk) roughly equated to the Rule of Six, work from home, and 10 pm rule, with the addition of closing businesses with music and dancing that opens at night. Tier 2 added no gatherings in indoor space between households, restricted travel, and increased the number of venues that closed. Tier 3 prevented meeting in private outdoor spacing with non-household members and restricted restaurants and bars to table service only, with serving of alcoholic drinks only allowed when consumed alongside a substantial meal [10].

The second national lockdown was less stringent than the first as schools remained open, but included closing of pubs, restaurants, gyms, and non-essential shops and asking people to stay at home [11].

### Study design

Our study is a longitudinal natural experiment. For each participant, we have one observation prior to the implementation of, and one observation after the restriction. Observations are at most 16 days from the date of the start of the restriction. This allows individuals within our study to be their own control and thus reduces the effect of between-person variation as well as the effect of longer-term temporal trends. The types of contact reported were categorised as home-based, work contact, school contact, and in other settings.

We compared the number of contacts before implementation of restrictions to the number of contacts after to assess the impact of (i) local restrictions; (ii) three national restrictions (1) Rule of Six, (2) 10 pm closure, and (3) work from home; (iii) entry into each of Tier 1, Tier 2, and Tier 3; and (iv) entry into the national lockdown from Tier 1, Tier 2, and Tier 3. To assess the effect of the different restrictions, we concentrated on changes in setting-specific contacts. For instance, local restrictions and the Tier system are largely targeted at leisure contacts, and the Rule of Six does not apply for businesses or schools. Hence, for these two restrictions, we analysed changes in contacts excluding work and school. The 10 pm closure rule requires restaurants, pubs, and bars to close early, and is therefore not expected to have a direct effect on contacts made at home, work, or school. Thus, we excluded contacts in those settings as the outcome for this restriction and refer to remaining contacts as *Other* contacts. To assess the effect of the work from home advice, we focused on the work contacts of respondents who were employed. During the second national lockdown, schools remained open, and therefore, we only excluded contacts made at school in assessing its effect.

### Statistical analysis

R version 4.0.0 was used for all analyses and the code and data are available on github (see the “Availability of data and materials” section) [12–14]. Descriptive and graphical summaries of participant characteristics for age, gender, employment, and socio-economic status were created for each restriction, for the change in mean contacts, and for the spatial and temporal variation in the restrictions. Uncertainty for the mean contacts was calculated using clustered bootstrapping [15] where sampling was done per person rather than per observation level to preserve the correlation structure of the data.

We compared contacts before and during restrictions by calculating the mean, median, and interquartile range (IQR). The change in contacts was categorised into increased, same (unchanged), and decreased. We calculated the mean of the paired differences in contacts before and after restrictions and assessed uncertainty by constructing a 95% confidence interval (95% CI) from 10,000 bootstrap samples [15] of the paired differences.

For each restriction, we conducted paired permutation tests [16] with 50,000 permutations per test. We chose permutation tests as they are robust to distributional assumptions of the underlying data [14]. In order to preserve the study structure, we calculated the paired difference by subtracting the reported number of contacts during the restriction from the reported number of contacts before the restriction and then randomly changed the sign of each pair. In practice, this means generating a vector of random values taking − 1 and 1 that is of the same length as the number of participants and then multiplying the change in contacts by this vector.

We decided to calculate two test statistics for each permutation and each restriction: (1) the proportion of individuals whose contacts decreased after restrictions were implemented, and (2) the mean of the change in contacts before and after restrictions. The proportion of decreases is robust to large values and skewed distributions treating a difference of − 1 and − 1000 in the same way. This measure tests the relative effect of the restriction but does not estimate the effect size. The mean difference estimates the absolute effect but is affected by skewed data. In order to reduce the impact of the skewness, we restricted the total number of contacts to 200 per person per day for the comparison of the means only.

We conducted further assessment of the restrictions by age group for the Rule of Six and the 10 pm rule as these restrictions are likely to have greater potential of an effect in younger individuals who are more likely to be mobile, asymptomatic if infected, and not be shielding. These analyses were stratified by age groups 5–17, 18–39, 40–59, and 60 +.

## Results

### Participant characteristics

There were 3884 participants included in the analysis for the Rule of Six: 3887 for 10 pm closure, 1408 for work from home, and 572 participants for local restrictions (Table 1). There were 2415 entering into Tier 1, 1654 entering Tier 2, and 368 entering Tier 3. Furthermore, there were 2095 leaving Tier 1 into national lockdown, 1445 leaving Tier 2 into national lockdown, and 323 leaving Tier 3 into national lockdown. The age distributions of the samples for Rule of Six, 10 pm closure, local restrictions, entry to tiers, and national lockdown were similar with over 30% of the samples being between 50 and 69 years of age in all nine analyses. The work from home category by definition only included participants 18 years of age or older and nearly 70% of participants were between 30 and 59. The gender split was close to 50% for all restrictions. Excluding the work from home analysis, around 40% of participants were employed for each restriction (which reflects the broad age range of the sample, including children and the elderly). Socio-economic status was consistent across each analysis samples with lowest numbers in the A - upper middle class, and E - lower level of subsistence categories and the modal group being C1 - lower middle class for all restrictions (Table 1).

**Table 1:** Participants characteristics in the CoMix survey for each restriction

Restrictions					Entry into			Exit from tier to lockdown		
	Rule of Six	10 pm closure	Work from home	Local lockdown	Tier 1	Tier 2	Tier 3	Tier 1	Tier 2	Tier 3
	*N* (col %)	*N* (col %)	*N* (col %)	*N* (col %)	*N* (col %)	*N* (col %)	*N* (col %)	*N* (col %)	*N* (col %)	*N* (col %)
**Total**	3884	3887	1408	572	2415	1654	368	2095	1455	323
**Age groups**										
0–4	129 (3.3%)	142 (3.7%)	0	22 (3.9%)	96 (4.0%)	65 (4.0%)	12 (3.3%)	53 (2.5%)	49 (3.4%)	13 (4.0%)
5–11	214 (5.5%)	275 (7.1%)	0	54 (9.5%)	151 (6.3%)	117 (7.1%)	36 (9.9%)	120 (5.8%)	85 (5.9%)	31 (9.7%)
12–17	261 (6.8%)	291 (7.5%)	0	59 (10.4%)	202 (8.4%)	121 (7.4%)	48 (13.2%)	149 (7.1%)	101 (7.0%)	41 (12.8%)
18–29	364 (9.4%)	384 (9.9%)	222 (15.8%)	61 (10.7%)	197 (8.2%)	161 (9.8%)	23 (6.3%)	158 (7.6%)	124 (8.6%)	20 (6.2%)
30–39	462 (12.0%)	432 (11.2%)	308 (21.9%)	65 (11.4%)	240 (10.0%)	191 (11.6%)	42 (11.6%)	194 (9.3%)	153 (10.6%)	34 (10.6%)
40–49	495 (12.8%)	531 (13.7%)	363 (25.8%)	89 (15.6%)	326 (13.6%)	219 (13.3%)	47 (12.9%)	288 (13.8%)	200 (13.8%)	42 (13.1%)
50–59	708 (18.3%)	613 (15.8%)	322 (22.9%)	90 (15.8%)	402 (16.7%)	277 (16.9%)	63 (17.4%)	355 (17.0%)	262 (18.1%)	49 (15.3%)
60–69	723 (18.7%)	751 (19.4%)	174 (12.4%)	88 (15.5%)	449 (18.7%)	309 (18.8%)	50 (13.8%)	428 (20.5%)	291 (20.1%)	48 (15.0%)
70+	506 (13.1%)	449 (11.6%)	19 (1.3%)	41 (7.2%)	341 (14.2%)	181 (11.0%)	42 (11.6%)	341 (16.3%)	182 (12.6%)	43 (13.4%)
Missing	22	19	–	3	11	13	5	9	8	2
**Gender**										
Female	2013 (52.0%)	2004 (51.6%)	718 (51.1%)	277 (48.7%)	1252 (52.0%)	890 (54.0%)	179 (48.8%)	1072 (51.3%)	758 (52.2%)	156 (48.4%)
Male	1861 (48.0%)	1877 (48.4%)	688 (48.9%)	292 (51.3%)	1156 (48.0%)	759 (46.0%)	188 (51.2%)	1018 (48.7%)	694 (47.8%)	166 (51.6%)
Missing	10	6	2	3	7	5	1	5	3	1
**Employed**										
Yes	1487 (38.3%)	1433 (36.9%)	1408 (100.0%)	220 (38.5%)	882 (36.5%)	608 (36.8%)	134 (36.4%)	761 (36.3%)	521 (35.8%)	112 (34.7%)
No	2397 (61.7%)	2454 (63.1%)	0	352 (61.5%)	1533 (63.5%)	1046 (63.2%)	234 (63.6%)	1334 (63.7%)	934 (64.2%)	211 (65.3%)
**Socio-economic status**										
A - Upper middle class	200 (5.1%)	214 (5.5%)	72 (5.1%)	24 (4.2%)	143 (5.9%)	89 (5.4%)	14 (3.8%)	119 (5.7%)	79 (5.4%)	9 (2.8%)
B - Middle class	1061 (27.3%)	1033 (26.6%)	394 (28.0%)	161 (28.1%)	622 (25.8%)	418 (25.3%)	90 (24.5%)	554 (26.4%)	375 (25.8%)	85 (26.3%)
C1 - Lower middle class	1285 (33.1%)	1332 (34.3%)	536 (38.1%)	184 (32.2%)	812 (33.6%)	596 (36.0%)	130 (35.3%)	731 (34.9%)	529 (36.4%)	115 (35.6%)
C2 - Skilled working class	534 (13.7%)	529 (13.6%)	197 (14.0%)	85 (14.9%)	343 (14.2%)	227 (13.7%)	50 (13.6%)	278 (13.3%)	206 (14.2%)	40 (12.4%)
D - Working class	571 (14.7%)	556 (14.3%)	203 (14.4%)	82 (14.3%)	377 (15.6%)	221 (13.4%)	55 (14.9%)	304 (14.5%)	176 (12.1%)	47 (14.6%)
E - Lower level of subsistence	233 (6.0%)	223 (5.7%)	6 (0.4%)	36 (6.3%)	118 (4.9%)	103 (6.2%)	29 (7.9%)	109 (5.2%)	90 (6.2%)	27 (8.4%)

### All contacts in adults and restrictions

**Fig. 1:**
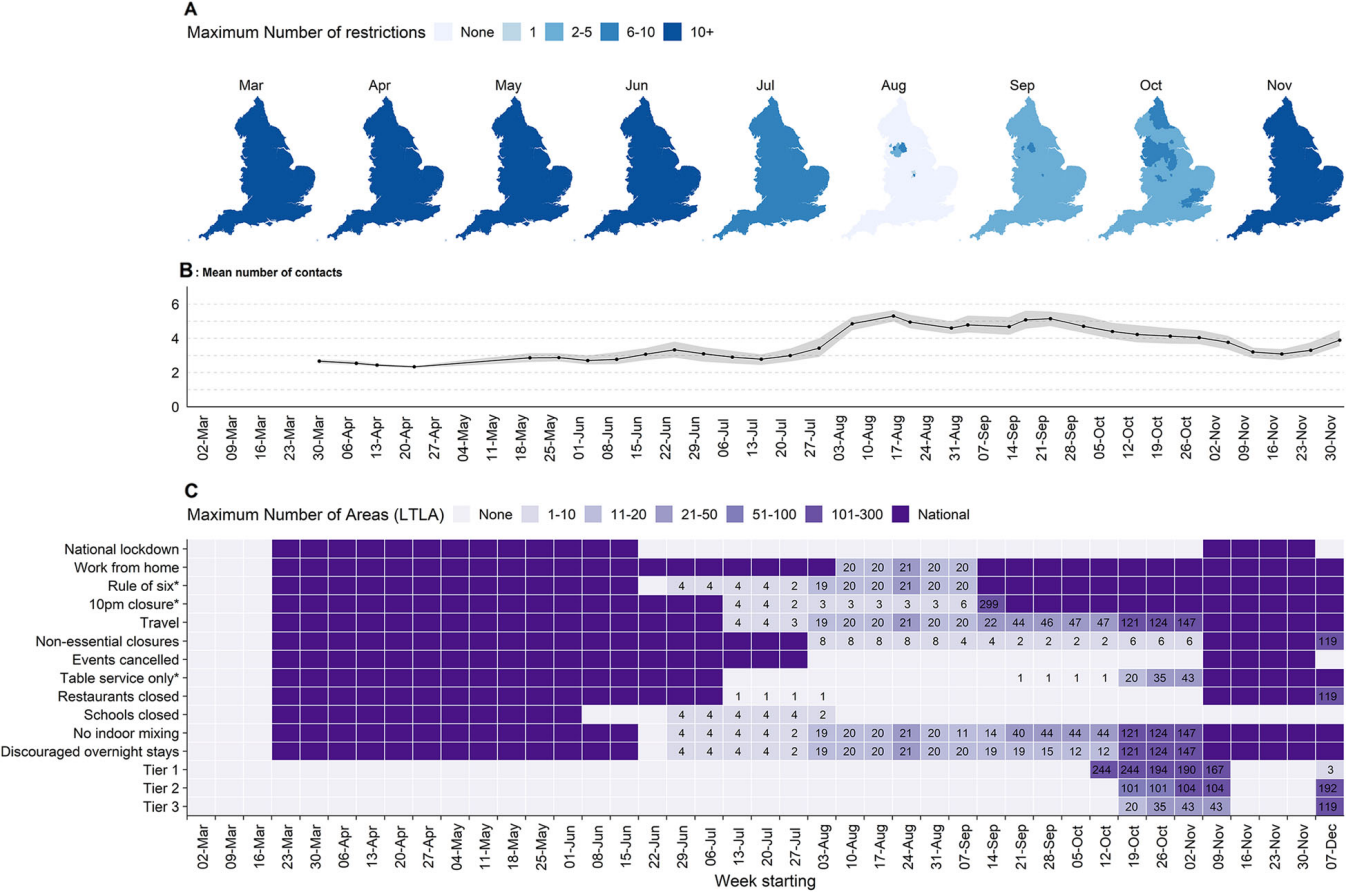
**a** Number of restrictions by English Lower Tier Local Authority (LTLA) over time. **b** Mean contacts for adults from CoMix over time. **c** Heatmap of number of LTLA areas affected by each restriction over time. Graph A shows the geographical distribution of the restriction by LTLA region in England overtime. The number of restrictions represents the maximum number of restrictions applied at any time during a single month. Graph B shows the mean number of contacts in all settings with a cap of 200 contacts per person, 2000 bootstrap samples were used to generate 95% uncertainty intervals with sampling done at the participant level to reflect clustering of repeated measures. Graph C shows a temporal heatmap of the number of LTLA areas that were affected by each restriction. *Rule of Six and 10 pm rules are included in the first national lockdown as more stringent rules were applied with all restaurants and bars being closed and no meetings allowed with any other households

Restrictions were applied nationally in England from March through to June (Fig. 1). In the summer, restrictions were reduced, and local restrictions were applied relating to travel, non-essential closures, no indoor mixing, and discouraging overnight stays (Fig. 1c). These restrictions were mostly applied in the North of England (Fig. 1a). The relaxing of restrictions in August coincides with an increase in the mean contacts for adults, with contacts gradually reducing again from September to November as restrictions became more stringent and widespread (Fig. 1b). Following the second national lockdown, mean daily reported contacts returned to similar levels as in July. Figure 1 presents mean contacts over time for adults only, as data on children were not collected throughout this period of the survey, and as the 2nd national lockdown did not involve school closure.

### Distribution of setting-specific contacts

**Fig. 2:**
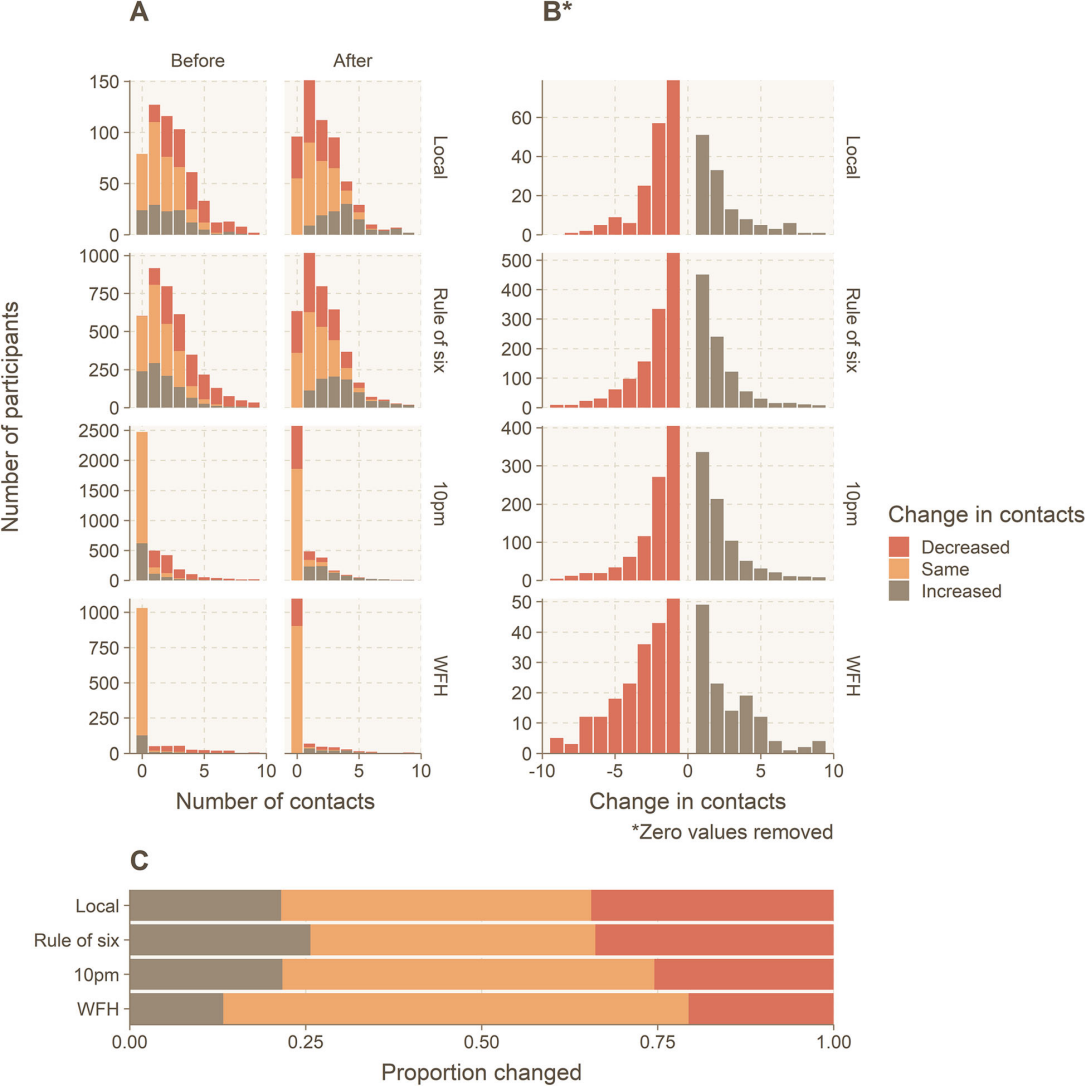
**a** The distribution of the number of setting-specific contacts before and after each restriction came into place. **b** Change in contacts for each restriction. **c** The proportion of changes comparing before and after the restrictions started. Graph A shows the distribution of setting-specific contacts before and after each restriction came into place. Colours represent whether the change in contacts increased (brown), decreased (yellow), or stayed the same (orange). Values greater than 10 are not shown on the graph. Graph B shows the distributions of the difference in contacts, with zero values removed to ease comparison between increases and decreases. Differences of magnitude greater than 10 are not shown. Graph C shows the proportions of changes in contacts due to each restriction. For most participants, the number of contacts remained unchanged (yellow) after restrictions. Additional file 1 Figure S1A and S1B show graphs 2A and 2B without the restrictions on the axes and with zero values included

**Fig. 3:**
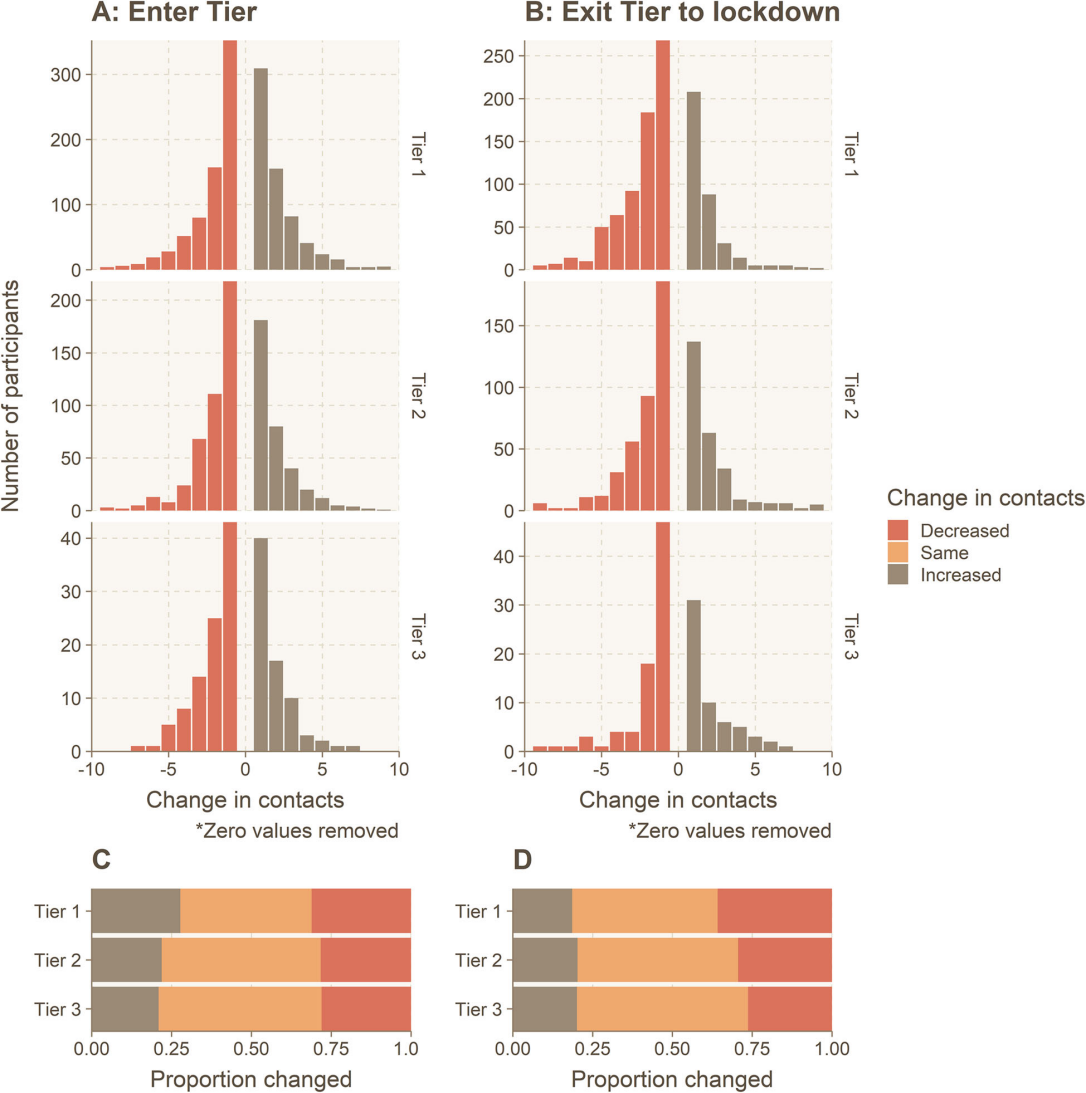
**a** Change in contacts after entry into each tier. **b** Change in contacts after entry into national lockdown from each tier. **c** Proportion of changes in contacts after entry into each tier. **d** Proportion of changes in contacts after entry into lockdown from each tier. Graph A and B show the distributions of the difference in contacts. Zero values are not shown to ease comparison between increases and decreases. Differences of magnitude greater than 10 are not shown. Graph C and D show the proportion of change in contacts due to each restriction. For the majority of people, the number of contacts remained unchanged after entry into each tier or exit from each tier to lockdown. Additional file 2 Figure S2A and S2B show graphs 3B and 3B without the restrictions on the axes and with zero values included

The setting-specific contacts were positively skewed for all restrictions (Fig. 2a, Table 2). The Rule of Six and local restrictions had similar distributions with the modal response being one contact before and the restrictions, whereas work from home and the 10 pm rule had a majority of individuals reporting zero contacts. The distributions for entry to tiers and exit from tiers to lockdown were similar with a median of 2 and IQR of 1 to 3, excluding exit tier 1 to lockdown, which had an IQR of 1 to 4. Overall, the magnitudes of the change in contacts were small, and for most individuals, the number of reported contacts did not change after each measure was introduced (Table 2). In order to show the patterns in the data, we restricted the axes and removed zero values in Figs. 2b, 3a, b. These graphs are reproduced in the Additional file 1: Figure S1A and S1B and Additional file 2: Figure S2A and S2B without removal of zero values or restriction of axes for comparison.

**Table 2:** Summary of permutation test on the proportion of individuals with decreased contacts and paired mean difference before and after restrictions

**Comparison with proportion decreased**								
**Restriction**	**Contacts**	***N***	**Adults**	**Children**	**Decreased**	**Same**	**Increased**	***P*** **value**
Local	exclude work and school	572	434	138	197 (34.4%)	252 (44.1%)	123 (21.5%)	< 0.001
ROS	exclude work and school	3884	3258	626	1314 (33.8%)	1573 (40.5%)	997 (25.7%)	< 0.001
10 pm	other	3887	3160	727	990 (25.5%)	2054 (52.8%)	843 (21.7%)	< 0.001
WFH	work	1408	1408	0	288 (20.5%)	933 (66.3%)	187 (13.3%)	< 0.001
T1 entry	exclude work and school	2415	1955	460	752 (31.1%)	993 (41.1%)	670 (27.7%)	0.017
T2 entry	exclude work and school	1654	1338	316	468 (28.3%)	823 (49.8%)	363 (21.9%)	< 0.001
T3 entry	exclude work and school	368	267	101	103 (28.0%)	188 (51.1%)	77 (20.9%)	0.034
T1 exit to LD	exclude school	2095	1764	331	750 (35.8%)	955 (45.6%)	390 (18.6%)	< 0.001
T2 exit to LD	exclude school	1455	1212	243	428 (29.4%)	732 (50.3%)	295 (20.3%)	< 0.001
T3 exit to LD	exclude school	323	236	87	85 (26.3%)	173 (53.6%)	65 (20.1%)	0.062
**Comparison in mean difference**		**Median (IQR)**			**Mean**			
**Restriction**	**Contacts**	**Before**	**After**		**Before**	**After**	**Difference (95% CI)**	***P*** **value**
Local	Exclude work and school	2 (1 to 4)	2 (1 to 3)		3.18	2.49	− 0.69 (− 1.25 to − 0.17)	0.004
ROS	Exclude work and school	2 (1 to 3)	2 (1 to 3)		2.9	2.66	− 0.25 (− 0.5 to − 0.01)	0.045
10 pm	Other	0 (0 to 1)	0 (0 to 1)		1.37	1.38	0.01 (− 0.23 to 0.23)	0.915
WFH	Work	0 (0 to 1)	0 (0 to 0)		4.62	3.62	− 0.99 (− 1.94 to − 0.03)	0.042
T1 entry	Exclude work and school	2 (1 to 3)	2 (1 to 3)		2.79	2.66	− 0.13 (− 0.39 to 0.11)	0.305
T2 entry	Exclude work and school	2 (1 to 3)	2 (1 to 3)		2.42	2.56	0.14 (− 0.17 to 0.55)	0.473
T3 entry	Exclude work and school	2 (1 to 3)	2 (1 to 3)		3.09	2.32	− 0.77 (− 1.97 to − 0.03)	0.067
T1 exit to LD	Exclude school	2 (1 to 4)	2 (1 to 3)		4.21	2.81	− 1.40 (− 2.03 to − 0.85)	< 0.001
T2 exit to LD	Exclude school	2 (1 to 3)	1 (1 to 3)		3.4	2.97	− 0.42 (− 1.13 to 0.33)	0.247
T3 exit to LD	Exclude school	2 (1 to 3)	2 (1 to 3)		3.08	3.54	0.46 (− 0.28 to 1.41)	0.343

Two-sided *p* value calculated by counting the number of permutations where the magnitude of the test statistics is greater than the observed test statistics, and dividing by the number of permutations

*ROS* Rule of Six, *WFH* encouraged to work from home, *LD* lockdown, *T1* Tier 1, *T2* Tier 2, *T3* Tier 3, *IQR* inter-quartile range

### National restrictions

#### Rule of Six

We compared non-work and non-school contacts for 3884 individuals before and after the Rule of Six came into effect. There was very strong evidence (*p* < 0.001) to suggest that more people reduced their contacts (excluding work and school) following the Rule of Six restriction than expected due to chance, with 1314 (33.8%) recording fewer contacts compared to 997 (25.7%) recording a greater number of contacts. However, in most participants, 1573 (40.5%) recorded the same number of contacts and the median number of contacts was 2 (IQR 1 to 3) before and after the introduction of the measure. There was a slight suggestion (*p* = 0.05) of a small reduction (− 0.25; − 0.5 to − 0.01) of mean non-work and non-school contacts per day (Table 2). Age-stratified analysis (Table 3) suggests that the Rule of Six had the greatest effect on the contact patterns of young adults (18–39 years), who reduced non-work and non-educational contacts by a mean of − 0.59 (− 1.09 to − 0.04).

**Table 3:** Summary of permutation test on the proportion of individuals with decreased contacts and paired mean difference before and after 10 pm and Rule of Six restrictions by age

**Comparison with proportion decreased**								
**Restriction**	**Contacts**	***N***	**Adults**	**Children**	**Decreased**	**Same**	**Increased**	***P*** **value**
**ROS**								
5–17	Exclude work and school	464	0	464	167 (36%)	179 (38.6%)	118 (25.4%)	0.0022
18–39	Exclude work and school	816	816	0	291 (35.7%)	343 (42%)	182 (22.3%)	< 0.001
40–59	Exclude work and school	1193	1193	0	396 (33.2%)	488 (40.9%)	309 (25.9%)	0.0005
60+	Exclude work and school	1225	1225	0	403 (32.9%)	475 (38.8%)	347 (28.3%)	0.0219
**10 pm**								
5–17	Exclude work and school	550	0	550	167 (30.4%)	239 (43.5%)	144 (26.2%)	0.1062
18–39	Exclude work and school	813	813	0	243 (29.9%)	376 (46.2%)	194 (23.9%)	0.0103
40–59	Exclude work and school	1134	1134	0	350 (30.9%)	507 (44.7%)	277 (24.4%)	0.002
60+	Exclude work and school	1196	1196	0	395 (33%)	453 (37.9%)	348 (29.1%)	0.0452
**Comparison in mean difference**		**Median (IQR)**			**Mean**			
**Restriction**	**Contacts**	**Before**	**After**		**Before**	**After**	**Difference (95% CI)**	***P*** **value**
**ROS**								
5–17	Exclude work and school	3 (2 to 4)	3 (2 to 4)		3.93	4.09	0.17 (− 0.35 to 0.76)	0.5668
18–39	Exclude work and school	2 (1 to 3)	2 (1 to 3)		2.89	2.3	− 0.59 (− 1.09 to − 0.04)	0.0183
40–59	Exclude work and school	2 (1 to 3)	2 (1 to 3)		2.61	2.31	− 0.3 (− 0.67 to 0.03)	0.1055
60+	Exclude work and school	2 (1 to 3)	2 (1 to 3)		2.56	2.6	0.04 (− 0.39 to 0.52)	0.8797
**10 pm**								
5–17	Exclude work and school	3 (2 to 4)	3 (2 to 4)		3.85	4.84	0.98 (0.28 to 1.81)	0.0115
18–39	Exclude work and school	2 (1 to 3)	2 (1 to 3)		2.7	2.57	− 0.13 (− 0.72 to 0.5)	0.649
40–59	Exclude work and school	2 (1 to 3)	1 (1 to 3)		2.4	2.54	0.14 (− 0.37 to 0.65)	0.6183
60+	Exclude work and school	2 (1 to 3)	1 (1 to 3)		2.54	2.09	− 0.45 (− 0.88 to − 0.12)	0.0039

Two-sided *p* value calculated by counting the number of permutations where the magnitude of the test statistics is greater than the observed test statistics, and dividing by the number of permutations

*ROS* Rule of Six, *IQR* inter-quartile range

#### Ten pm closure

We compared ‘other’ contacts (excluding home, work, or school) among 3887 participants before and after the 10 pm closure. There was very strong evidence (*p* < 0.001) to suggest that more people reduced their contacts following the 10 pm rule than expected due to chance, with 990 (25.5%) recording fewer contacts compared to 843 (21.7%) recording a greater number of contacts. However, more than half of the participants 2054 (52.8%) recorded the same number of contacts and the median number of contacts was very low (0; IQR 0 to 1) before and after the 10 pm rule. The data were consistent with no absolute effect (*p* = 0.915) with the change in mean ‘other’ contacts estimated as 0.01 (− 0.23 to 0.23) (Table 2). Subgroup analysis suggested inconsistent patterns by age group (Table 3), which might be expected if overall there is no evidence of changes in contacts following this measure.

#### Work from home

Over two thirds of participants 933 (66.3%) had the same number of work contacts before and after being encouraged to work from home. Despite this, the data strongly suggest (*p* = 0.001) that a greater number reduced their work contacts after the restriction came into place than would be expected due to chance. Differences in work contacts were highly skewed with forty participants reporting a difference of more than 50 contacts, yet the 25th and 50th quantile of the difference being zero (Fig. 2, Table 2). The data were compatible (*p* = 0.05) with a reduction in the mean work contacts. Though there was large uncertainty around the point estimate, it was far from zero (− 0.99 contacts per day, 95% CI − 1.94 to − 0.03; Table 2).

#### Local restrictions

There was strong evidence (*p* < 0.001) that following local restrictions more participants reduced their non-work and non-school contacts than would be expected due to chance. Of the 572 participants, 197 (34.4%) individuals reported fewer contacts, 123 (21.5%) reported greater contacts, and 252 (44.1%) reported the same number of contacts. On average, participants reported 0.69 (0.17 to 1.25; *p* = 0.004) fewer non-work and non-school contacts compared to before the restrictions, corresponding to a relative reduction of 21% (5% to 40%).

#### Entry into tiers

We compared non-work and non-school contacts for 2415 individuals before and after entry into Tier 1, 1654 for Tier 2, and 368 for Tier 3 (Table 2). There was a strong suggestion that more people reduced their contacts than would be expected due to chance after entry into each tier, though there was a higher percentage of people who increased their contacts when entering into Tier 1 compared to entry in other levels. Indeed, the data were consistent with no reduction in mean contacts after entering into Tier 1 or Tier 2 with change in reported contacts close to zero. There was a suggestion of a reduction in daily contacts from 3.09 to 2.32 following entry into Tier 3 (*p* = 0.067), but there are only 368 observations in this category. The median number of daily contacts remained fixed at 2 (IQR 1 to 3) before and after entry into all tiers.

#### Exit from tiers into national lockdown

We compared non-school contacts for 2095 individuals before and during the national lockdown entering from Tier 1, 1455 entering from Tier 2, and 323 entering from Tier 3. The data were consistent with more individuals reducing their contacts than expected due to chance. The largest difference was observed for those entering lockdown form Tier 1 with 750 (35.8%) reducing their contacts versus 390 (18.6%) increasing them (Table 2). This was consistent with the strong evidence (*p* < 0.001) for a reduction of 1.40 (0.85 to 2.03) in the mean daily number of contacts when entering from Tier 1 to lockdown. The effect of moving from Tier 2 or Tier 3 to lockdown was less pronounced, though there were few observations for the estimate of Tier 3 to lockdown.

## Discussion

Along with many other countries, England transitioned from a national lockdown approach to more localised interventions with less restrictive national measures and subsequently reverted to a national lockdown in the autumn of 2020. We determine that the impact of these measures is mixed: the imposition of local measures (which were very varied in different places) and the Rule of Six probably led to modest reductions in contacts; instructions to work from home if possible led to a larger reduction in contact, whereas there is little to no evidence that the 10 pm closing time for bars and restaurants had any appreciable effect. The adoption of the tier system was similarly mixed, with Tier 1 and 2 having little impact on mean contacts, but Tier 3 (the most stringent) reducing the mean daily reported number of contacts. The subsequent imposition of the lockdown appears to have reduced contacts in those individuals who were previously under Tier 1 (the lightest restrictions), but the data is less supportive of an effect in individuals who were already under tighter restrictions (Tiers 2 and 3), potentially because as it was more difficult for these participants to further reduce their contacts.

In absolute terms, these changes in mean contacts are relatively small. However, the relatively small size of the absolute effects in this study may be more indicative that restrictions were applied at a point when individuals already had lowered their contacts, as opposed to an indication that the restrictions would not have an effect. For instance, the appeal to work from home only reduced the mean daily work contacts by about 1, though a larger sized effect would perhaps have been difficult to achieve as working from home was already relatively commonplace at the time the measure was implemented [17]. To put these changes in context, the full national lockdown in March reduced the average daily contacts from an estimated 10.8 to 2.8 [2]. This 74% reduction, in turn, reduced the effective reproduction number (*R*_0_) of COVID-19 from about 2.6 before lockdown to about 0.6 after lockdown [2]. The impact on *R*_0_ from the relatively small reduction in mean work contacts made under the various restrictions discussed here would likely have a much more marginal impact on *R*_0_.

Determining the epidemiological effect of restrictions has proved challenging. This is because of delays between the imposition of measures and their effect on reported cases, hospitalisations, and deaths. Furthermore, reported numbers of cases might be biased upwards in areas of local restrictions if additional efforts are put in place to find and test cases in these regions. Estimating the counterfactual—how many cases might have occurred without the restrictions—is also very difficult to do. For these reasons, evidence on the effect of local and national restrictions is weak. This study takes a different approach. Contacts might be expected to change immediately after restrictions are in place and would be less affected by changes in case finding. Furthermore, the longitudinal panel nature of the data allows for individuals to act as their own temporal control group, making it easier to pick up relatively small changes in contact patterns.

This work has several limitations. We had to group several types of measures used in local restrictions and therefore the effect that we see is a combination of a range of interventions. Individuals may also not accurately report their contacts, due to recall or social desirability bias. A further limitation is that restrictions were not randomly allocated and thus the effects seen may be due to other confounding factors. However, we did use a repeated measure on the same individuals, which will reduce between-person variability, though confounding factors could remain constant on individuals and affect the generalisability of results. The contact data is bounded at zero and skewed; therefore, using the mean can be a less relevant summary measure; this is why we also performed a permutation test that focused on the sign of the difference rather than the magnitude. Furthermore, we did not distinguish between the length of time spent with different contacts. Finally, as contacts decline, the possibility for individuals to further reduce their social interactions decreases. Any changes in contacts would therefore be expected to be small and would require a very large survey to quantify accurately.

Our study design—concentrating on contacts 16 days before and after new measures—attempted to reduce the effect of temporal trends in contacts. However, it is likely that it did not do this entirely. In addition, the relatively rapid change in policies over the autumn means that some of the effect attributed to one of the interventions may have actually been caused by one of the others. We looked at setting specific contacts (e.g. work and school) to try to limit these potential spill-overs but cannot be sure that we eliminated them entirely. A shorter period of study (e.g. 1 week before or after the new measures) would reduce both of these potential issues, but would also significantly reduce our sample size (as data is collected in alternating weeks), and therefore power to detect a difference.

Despite these limitations, we have attempted to provide insight into the highly relevant issue of whether different restrictions in response to COVID-19 work and if so, how effective they are. We only focused on one metric of epidemiological relevant setting-specific contacts, though the impact of the different restrictions will have broader societal implications that need to be considered for policy change.

Future work could assess whether restrictions reduce the amount of time spent with individuals, as may well be the case for the 10 pm rule. Further exploration of the effect of restrictions on different age groups and the potential of regional adherence to the national restrictions could help disentangle whether lack of effects was due to sampling biases rather than lack of effectiveness of restrictions.

## Conclusions

We have demonstrated that behavioural monitoring can allow the rapid evaluation of the impact of national and local restrictions on COVID-19 transmission. Although many of these restrictions appear to have led to behavioural change, the magnitude of these changes appears to be small.

## Supplementary Information

**Additional file 1: Figure S1A.** A: The distribution of the number of setting-specific contacts before and after each restriction came into place. **Figure S1B.** Change in contacts for each restriction.

**Additional file 2: Figure S2A.** Change in contacts after entry into each tier. **Figure S2B.** Change in contacts after entry into national lockdown from each tier.

## Data Availability

The code and data used to conduct these analyses are found at https://github.com/jarvisc1/comix_uk_covid_restrictions

## Abbreviations

CI: Confidence interval
IQR: Interquartile range
UK: United Kingdom
