# Supplementary figures and images for: The impact of local and national restrictions in response to COVID-19 on social contacts in England: a longitudinal natural experiment
Source: BMC Med. 2021 Feb 19;19:52. doi: 10.1186/s12916-021-01924-7 (PMC7892289; doi:10.1186/s12916-021-01924-7)

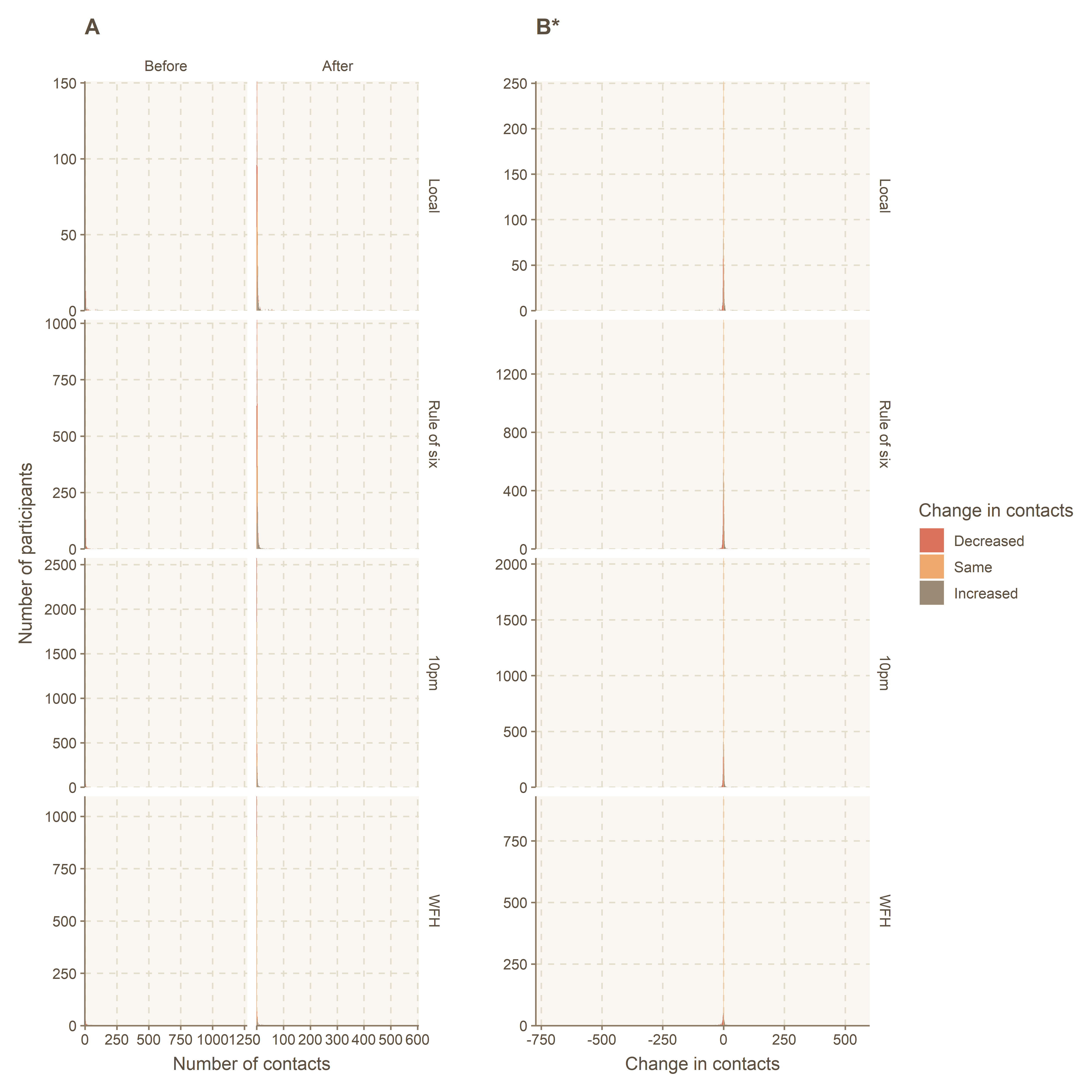

Supplement: Supplementary file 1 — Additional file 1: Figure S1A. A: The distribution of the number of setting-specific contacts before and after each restriction came into place. Figure S1B. Change in contacts for each restriction. [file 12916_2021_1924_MOESM1_ESM.png]

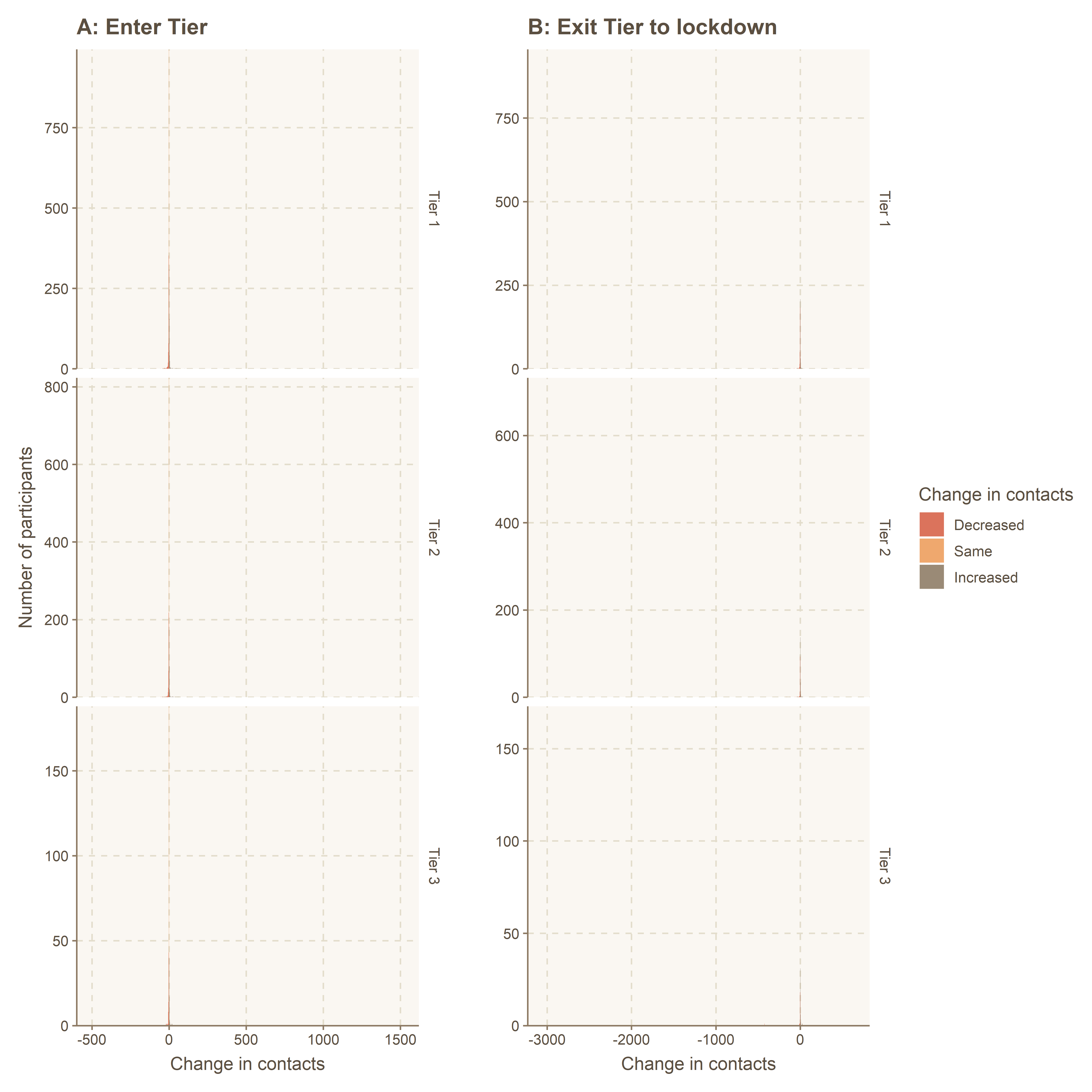

Supplement: Supplementary file 2 — Additional file 2: Figure S2A. Change in contacts after entry into each tier. Figure S2B. Change in contacts after entry into national lockdown from each tier. [file 12916_2021_1924_MOESM2_ESM.png]
